# Erratum

**DOI:** 10.1111/acel.12457

**Published:** 2016-09-06

Sriram, S., Subramanian, S., Sathiakumar, D., Venkatesh, R., Salerno, M. S., McFarlane, C. D., Kambadur, R. and Sharma, M. (2011), Modulation of reactive oxygen species in skeletal muscle by myostatin is mediated through NF‐κB. Aging Cell, 10: 931–948. doi: 10.1111/j.1474‐9726.2011.00734.x

In the article, we have inadvertently left out some specific details relating to how some of the figures were derived. To improve the clarity of the legends, we have included more details on how each of the figures was derived. These changes do not affect the conclusions of the study.

The correct legends to Figs  4, 6, and 8 are as follows:

**Figure 4:**
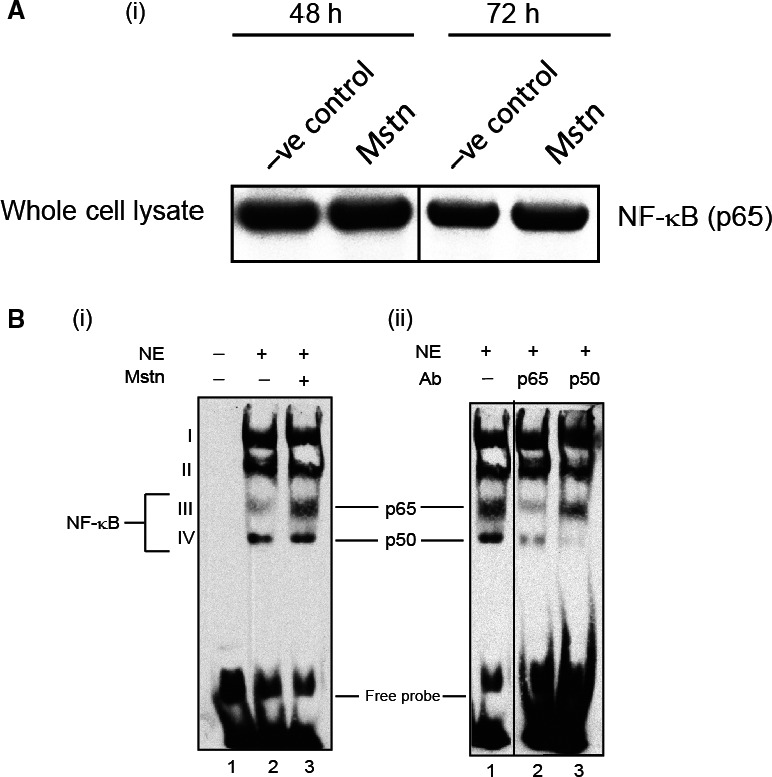
Myostatin (Mstn) induces reactive oxygen species (ROS) through tumor necrosis factor‐α (TNF‐α) via NF‐κB signaling. (A) Effect of Mstn on NF‐κB and IκB‐α in proliferating C2C12 myoblasts. Western blotting analysis was performed on whole cell lysates, nuclear and cytoplasmic extracts obtained from C2C12 cells treated with Mstn (3.5 ng mL^−1^) for indicated time points. (i) Left panel: representative immunoblot showing protein levels of NF‐κB (p65), p‐NF‐κB (p65), IκB‐α, p‐IκB‐α, and IKKα in negative control (lanes 1 and 3) and Mstn (lanes 2 and 4)‐treated whole protein lysates, nuclear and cytoplasmic extracts at indicated time points. α‐Tubulin was used as an internal control for equal protein loading on the gel. Other lanes from the representative whole cell lysate NF‐κB (p65) blot in (i) were spliced out of the original image as not being relevant to the comparison illustrated here so that only pertinent lanes from the original blots are shown. The position of the splice is indicated by the vertical line between lanes 2 and 3. Right panel: corresponding densitometry analysis of (ii) p‐NF‐κB (p65) and (iii) p‐IκB‐α showing significant increase or decrease in protein content upon Mstn treatment (*P < 0.05, **P < 0.01) (*n* = 2). (B) Mstn‐mediated enhancement of NF‐κB binding activity on mouse TNF‐α promoter. Electrophoretic mobility shift assay was performed using nuclear extracts from proliferating C2C12 cells treated with Mstn (3.5 ng mL^−1^) for 48 h. (i) Left panel: showing the increased activity of NF‐κB upon Mstn treatment. Lane 1, oligo only; lane 2, untreated; lane 3, Mstn‐treated. Electrophoretic mobility shift assay was also performed with nuclear extracts preincubated with the specific antibody. (ii) Right panel: NF‐κB complexes containing p65 or p50 subunits. Lane 1, no antibody added; lane 2, p65‐specific antibody added; lane 3, p50‐specific antibody added (*n* = 2). The images in panels (i) and (ii) are derived from one representative gel. Lane 3 in panel (i) is also shown in lane 1 panel (ii) to allow for direct comparison. An empty lane between lanes 1 and 2 in panel (ii) was spliced out of the original image as indicated by the vertical black line.

**Figure 5:**
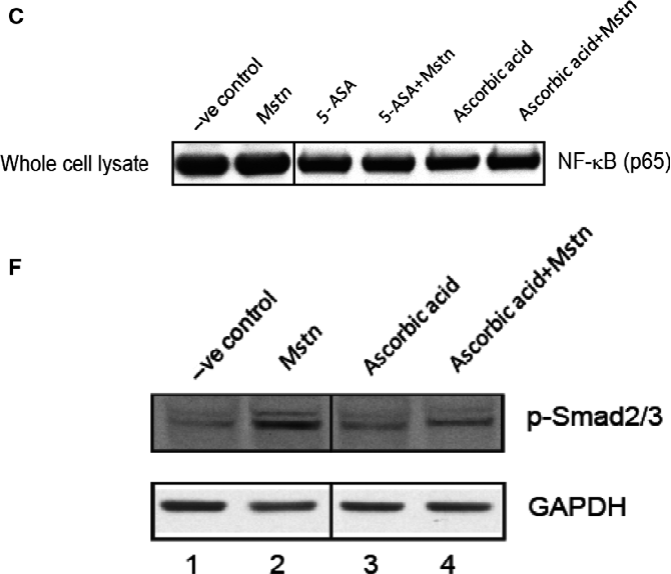
5‐ASA and ascorbic acid inhibit Mstn‐induced ROS via tumor necrosis factor‐α (TNF‐α) and NF‐κB signaling. mRNA expression analysis was performed on C2C12 cells treated with 5‐ASA (10 μm mL^−1^) and ascorbic acid (100 μm mL^−1^) along with Mstn (3 μg mL^−1^). Representative graphs show fold change in the mRNA expression of TNF‐α (A) and *Nox1* (B). The values are mean ± SE of two independent experiments. **P < 0.01 and ***P < 0.001 denote significant increase when compared to untreated cells; ^^*P* < 0.01 and ^^^*P* < 0.001 denote significant decrease when compared to Mstn‐treated cells (*n* = 2). (C) Western blotting analysis showing the level of NF‐κB‐p65 at 48‐h time point in whole cell lysates, nuclear and cytoplasmic extracts of proliferating C2C12 cells, untreated (lane 1), treated with Mstn (lane 2), 5‐ASA (lane 3), 5‐ASA and Mstn (lane 4), ascorbic acid (lane 5), and ascorbic acid and Mstn (lane 6). α‐Tubulin was used as an internal control for equal protein loading on the gel. The same image was used for lanes 1 and 2 of the NF‐κB (p65) blot for whole cell lysate in Fig. 5 (C) and lanes 1 and 2 in the NF‐κB (p65) blot for whole cell lysate in Fig. 4A (i). This required that lanes 1 and 2 be spliced into the image shown in 5C as indicated by the vertical black line. Other lanes from the representative whole cell lysate NF‐κB (p65) blot in (C) were excluded as not being relevant to the comparison being made, and only pertinent lanes from original blots are shown. Mstn induces the muscle‐specific E3 ligase expression via ROS‐dependent pathway. Western blotting analysis showing the levels of p‐Akt and Akt (48 h) (D), Atrogin1 and MuRF1 (72 h) (E) in the C2C12 cells, untreated (lane 1), treated with Mstn (3 μg mL^−1^) (lane 2), ascorbic acid (100 μm mL^−1^) (lane 3), and ascorbic acid and Mstn (lane 4, D) (*n* = 2). α‐Tubulin was used as an internal control for equal protein loading on the gels. (F) Representative gel showing p‐Smad2⁄3 protein levels in C2C12 cells, untreated (lane 1), treated with Mstn (3 μg mL^−1^) (lane 2), ascorbic acid (100 μm mL^−1^) (lane 3), and ascorbic acid and Mstn (lane 4) for 48 h during proliferation. GAPDH was used as an internal control for equal protein loading on the gel. Samples for Fig. 5F and Fig. 6D were run on the same gel. Irrelevant lanes from the representative p‐Smad2/3 and GAPDH blots in (F) were spliced out of the image as indicated by the vertical black line. Lanes 1 and 2 of Fig. 5F are the same as lanes 1 and 2 of Fig. 6D.

**Figure 6:**
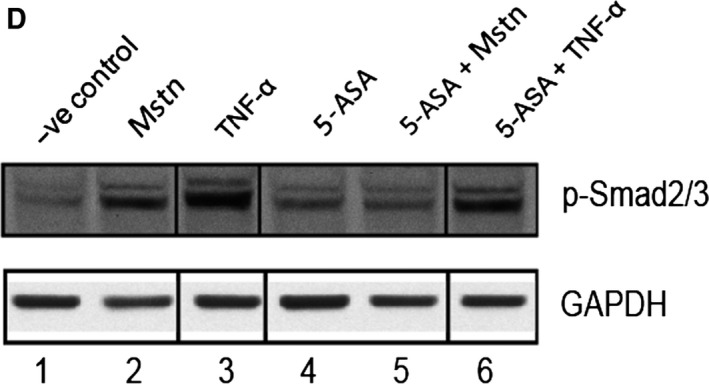
Tumor necrosis factor‐α (TNF‐α) induces myostatin (Mstn) expression, secretion, and signaling in a feed‐forward mechanism (A) Representative graph showing mRNA expression of *Mstn* and (B) representative gel showing protein levels of Mstn (~56 KDa‐full length) in C2C12 cells, untreated (lane 1), treated with TNF‐α (10 ng mL^−1^) (lane 2), 5‐ASA (10 μm mL^−1^) (lane 3), and 5‐ASA and TNF‐α (lane 4) for 72 h in proliferation media. The values are mean ± SE of two independent experiments. The level of significance was compared to the untreated control (****P < 0.0001) (*n* = 2). GAPDH was used as an internal control for equal protein loading on the gel. (C) The level of secreted Mstn in the cell culture media from C2C12 cells treated with TNF‐α (10 ng mL^−1^) and 5‐ASA (10 μm mL^−1^) was determined by EIA. The values are mean ± SE of two independent experiments. The concentration of Mstn is given as ng mL^−1^, and the level of significance was determined with respect to the negative control (**P < 0.01) (*n* = 2). (D) Representative gel showing p‐Smad2/3 protein levels in C2C12 cells, untreated (lane 1), treated with Mstn (3 μg mL^−1^) (lane 2), TNF‐α (10 ng mL^−1^) (lane 3), 5‐ASA (10 μm mL^−1^) (lane 4), 5‐ASA and Mstn (lane 5), and 5‐ASA and TNF‐α (lane 6) for 72 h during proliferation. GAPDH was used as an internal control for equal protein loading on the gel. Samples for Fig. 5F and Fig. 6D were run on the same gel. Irrelevant lanes from the representative p‐Smad2/3 and GAPDH blots in (D) were spliced out of the image as indicated by the vertical black lines. Lanes 1 and 2 of Fig. 5F are the same as lanes 1 and 2 of Fig. 6D.

**Figure 8:**
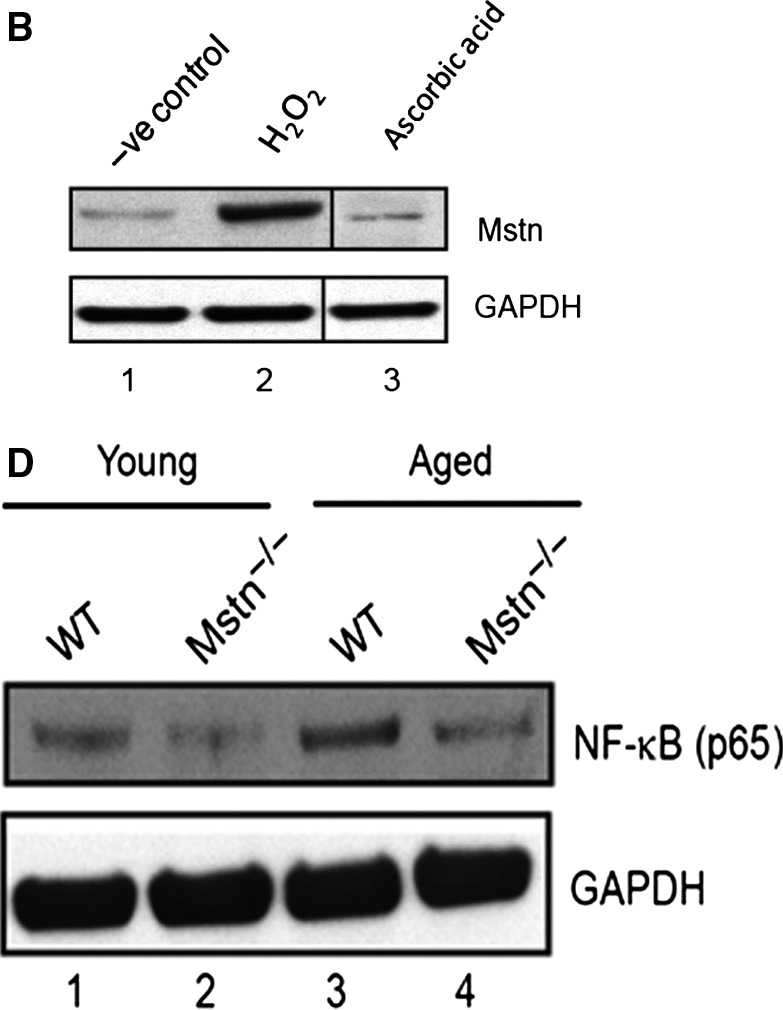
Hydrogen peroxide (H_2_O_2_) induces myostatin (Mstn) expression, secretion, and signaling (A) Representative graph showing mRNA expression of *Mstn* and (B) representative gel showing protein levels of Mstn in C2C12 cells, untreated (lane 1), treated with H_2_O_2_ (0.05 mm) (lane 2), and ascorbic acid (100 μm mL^−1^) (lane 3) for 72 h in proliferation media. The values are mean ± SE of two independent experiments. Significant increase (**P < 0.01) or decrease (***P < 0.001) in mRNA expression was compared to the untreated control (*n* = 2). GAPDH was used as an internal control for equal protein loading on the gel. Irrelevant lanes from the representative Mstn blot in (B) were spliced out of the image as indicated by the vertical black line. (C) The level of secreted Mstn in the cell culture media from C2C12 cells treated with H_2_O_2_ (0.05 mm) and ascorbic acid (100 μm mL^−1^) was determined by EIA. The values are mean ± SE of two independent experiments. The concentration of Mstn is given as ng mL^−1^, and the level of significance was determined with respect to the negative control (**P < 0.01) (*n* = 2). The absence of Mstn during aging decreases NF‐κB levels and increases AOE gene expression in skeletal muscle. (D) Western blotting analysis showing the level of NF‐κB‐p65 in protein lysates from Gas muscle of young (6‐week‐old) and aged (2‐year‐old) WT and Mstn^−⁄‐^ mice: young WT (lane 1), young Mstn^‐/‐^ (lane 2), aged WT (lane 3), and aged Mstn^‐⁄‐^ (lane 4). GAPDH was used as an internal control for equal protein loading on the gel. mRNA expression analysis in Gas muscle from young and aged WT and Mstn^‐⁄‐^ mice was performed. Representative graphs show fold change in the mRNA expression of *Sod1* (E) and *Cat* (F). The values are mean ± SE of three independent experiments. **P < 0.01, ***P < 0.001, and ****P < 0.0001 denote significant increase when compared to young and aged mice, and ^^^^P < 0.0001 denotes significant increase when compared to WT and Mstn^‐/‐^ mice (*n* = 3).

The internal control for Fig. 8D has been inadvertently shown as α‐tubulin in the manuscript, which has now been corrected with the appropriate internal control (GAPDH). The corrected figure is given below and the corrected legend is as above.

